# Comentario en torno al artículo “A propósito del bicentenario de la independencia de Colombia: las prácticas de lectura de Antonio Nariño y el desarrollo de una vacuna presuntamente efectiva contra la viruela”

**DOI:** 10.7705/biomedica.5723

**Published:** 2021-10-15

**Authors:** Esteban Vanegas

**Affiliations:** 1 Facultad de Medicina, Universidad de los Andes, Bogotá, D.C., Colombia Universidad de los Andes Facultad de Medicina Universidad de los Andes Bogotá D.C. Colombia

**Keywords:** viruela, virus de la viruela, vacuna contra viruela, inmunización, viru *Vaccinia*, epidemias, Smallpox, variola virus, smallpox vaccine, immunization, Vaccinia virus, epidemics

## Abstract

La viruela significó para las colonias americanas un proceso que desestabilizaba de forma dramática las dinámicas sociodemográficas de las colonias, lo que incentivó el desarrollo de estudios científicos sobre el virus causante. Cada libro acerca de la viruela en la biblioteca de Nariño constituyó una herramienta en la lucha contra el virus emprendida por el prócer. Tras la revisión del artículo “A propósito del bicentenario de la independencia de Colombia: las prácticas de lectura de Antonio Nariño y el desarrollo de una vacuna presuntamente efectiva contra la viruela” ^1^ quise comentar y profundizar en torno al saber médico de Nariño mediante el acercamiento a las obras a las que recurrió para instruirse sobre la enfermedad. A partir de la semblanza de cada una de ellas, analicé el proceso de variolización en el Reino de la Nueva Granada y la necesidad de fabricar una vacuna propia.

## Abstract (en)

For the American colonies, smallpox implied a process that dramatically destabilized their sociodemographic dynamics, which explains why scientific development took place around the causative virus. Each book about smallpox in Nariño’s library was a tool in the fight against smallpox undertaken by the founding father. After reviewing the article “About the bicentennial of the independence of Colombia: The reading practices of Antonio Nariño and the development of a vaccine that is presumably effective against smallpox*”*^1^, I set myself to study Antonio Nariño’s medical knowledge further. Through the approach to the works that Nariño used to educate himself on smallpox and the development of a biographical sketch of each of them, I analyzed the process of variolization in the Kingdom of Nueva Granada and the need to manufacture a vaccine locally.

## La viruela en América y la variolización en el Reino de la Nueva Granada

Los grandes aliados de la conquista española en los territorios americanos fueron los agentes patógenos que cruzaban el Atlántico a bordo de sus embarcaciones ^2^. Distintas hubiesen resultado las correrías de los conquistadores en el suelo de las Indias sin el amparo de los microbios que, con mortandad ingente, se propagaban entre las poblaciones amerindias ^2^^,^^3^. La viruela arribó al continente a principios del siglo XVI y su expansión diezmó las poblaciones nativas del Caribe y de la América continental ^2^^,^^4^; para 1520, ya se había propagado desde La Española y Cuba al golfo de México ^3^. El asedio de Hernán Cortes a México-Tenochtitlán solo pudo lograrse cuando el coctel de viruela, fiebre tifoidea y disentería arrasó con los ejércitos indígenas ^3^^,^^4^. Se cree que la viruela mató a más de 3 millones de aztecas ^3^^,^^4^. La primera epidemia de viruela en los territorios del Reino de la Nueva Granada acaeció en el año 1558 y provocó más de 40.000 muertos ^5^. Posteriormente, las epidemias se repitieron regularmente con hondas implicaciones sociodemográficas ^5^. Se han documentado hasta 11 epidemias de viruela en el periodo colonial, ocurridas con interludios de 20 años, aproximadamente ^5^^,^^6^.

Francisco Cosme Bueno, médico del Virreinato del Perú, documentó que la primera variolización en Hispanoamérica ocurrió en la ciudad de Popayán, hacia 1738 ^7^, dato proveniente de unas pocas fuentes. Por su parte, el naturalista francés Charles Marie de La Condamine señaló que el primer episodio de variolización en Hispanoamérica ocurrió de forma aislada en Brasil, hacia el año 1727 ^7^^,^^8^. El historiador y médico Hugo Sotomayor-Tribín precisó en su obra “Viruela en Colombia, de la Real Expedición Filantrópica al Parque de Vacunación” cuál fue, en su opinión, el primer proceso de variolización documentado en la región ^5^. Durante la epidemia de viruela que se inició en Cartagena de Indias en 1756, el párroco de San Juan de Girón, Joseph Velásquez Zilillaga, inoculó secreciones de un contagiado en 400 personas, aproximadamente ^5^. Se dice que el método tuvo tanto éxito que solo se registró un deceso a causa de la viruela en su poblado durante la epidemia, en tanto que causó más de 40.000 muertes en el Reino ^5^.

La masificación de la variolización en Hispanoamérica tuvo obstáculos ^8^^-^^10^. En la España peninsular había una amplia resistencia entre los médicos y los académicos del Real Tribunal del Protomedicato, pues la consideraban un método ineficaz y peligroso ^8^^,^^10^. Hubo publicaciones que proclamaban la peligrosidad del método, teniendo en cuenta los riesgos implícitos del proceso, las posibles secuelas de la infección benigna y la potencial fuente de contagio que representaba la persona inoculada ^8^^-^^10^. Además, había objeciones de naturaleza religiosa ^9^^,^^10^. En su diario, Mutis critica esta posición afirmando que “la decisión del protomedicato de Madrid era contraria a la propagación de un saludable método para España” ^5^^,^^11^. Durante la epidemia de 1782, el sabio publicó el “Método general para curar viruelas” ^5^^,^^11^^,^^12^ en el que adaptaba las técnicas de variolización de Europa a las condiciones del Reino, teniendo en cuenta la escasez de médicos y las prácticas generalizadas de curanderos y chamanes ^12^. La publicación de Mutis no solo era una detallada explicación del método de variolización: contenía diversas normas de higiene personal y de los espacios ^5^^,^^11^^,^^12^, y constituyó uno de los primeros esfuerzos en salud pública en América ^13^.

Mutis emprendió la tarea de inocular a gran parte de la población del Nuevo Reino ^5^^,^^11^^,^^14^. Con el aval del virrey Caballero y Góngora, se inició una campaña para masificar el método entre los granadinos ^5^^,^^14^ mediante la distribución de volantes que defendían la inoculación a pesar de las objeciones religiosas y culturales que eran comunes entre la población ^14^^,^^15^. Esta difusión comunitaria del método permitió la inoculación de más de 1.700 habitantes de Santa Fe hacia 1782 ^15^. El impacto de la variolización en ese año fue limitado: se produjo el deceso del 20 % de la población santafereña, pero en la epidemia de 1802, la tasa de mortalidad se redujo ostensiblemente (solo ocurrieron 300 muertes) ^12^^,^^13^.

## Las obras de Antonio Nariño en torno a la viruela

Ante la escasez de médicos en la Nueva Granada, Antonio Nariño se dedicó a atender a las personas pobres y a prescribir medicamentos durante las epidemias. Nariño era un defensor a ultranza de la variolización. Como lo señalan Moreno, *et al.*, Nariño poseía en su biblioteca tres obras médicas sobre la viruela ^1^^,^^5^, de las cuales hago a continuación una breve semblanza.

La “Disertación físico-médica en la cual se prescribe un método seguro para preservar a los pueblos de viruelas hasta lograr la completa extinción de ellas en todo el reino” es una obra del médico español Francisco Gil ^16^. La obra tiene una estrecha relación con el trabajo del prócer quiteño Eugenio Espejo “Reflexiones acerca de un método para preservar a los pueblos de las viruelas” ^17^, la cual constituye una clara manifestación humanista e ilustrada que sirvió de referencia y base para la disertación del médico español ^17^^,^^18^. Es tal la influencia del pensamiento del quiteño en la obra de Gil, que este último insertó el trabajo del prócer como apéndice de su libro en la segunda edición ^19^.

Para Espejo, la inoculación es un acto patriota que busca la conservación y la propagación del género humano para garantizar que los pobladores de una nación permanezcan ilesos ^17^^-^^19^. Espejo contraría la noción religiosa que tildaba la variolización como un acto pecaminoso que ponía en riesgo la integridad de los siervos de Dios ^17^^-^^19^. La variolización en manos del Estado se convertía en una herramienta humanitaria y se legitimaba como un proceder cristiano ^18^. Para José Celestino Mutis, Antonio Nariño y Eugenio Espejo, el poder del Estado radica en su modo de actuar frente a los riesgos que ponen en peligro a las clases trabajadoras que sustentan el desarrollo y el crecimiento social, político y económico ^20^.

A pesar de esta premisa de la obra criolla en la que se fundamentó el libro del español, este tenía un pensamiento más conservador ^16^, pues, si bien defendía la variolización, propendía por llevarla a cabo con mesura y prudencia ^16^. Enumeró de forma detallada sus riesgos y probables consecuencias, promoviendo su implementación únicamente por parte de médicos entrenados y convocando al Estado a que creara instituciones cuyo único fin fuera la inoculación y el aislamiento del inoculado ^16^. Gil rechazaba la inoculación sin las precauciones debidas y en su disertación enunció extensamente los riesgos de un proceso mal hecho ^16^. Para él, el paso fundamental de una variolización efectiva era el aislamiento del inoculado. Por esta razón defendía la creación de hospitales en las periferias de las ciudades dedicados exclusivamente a inocular ^16^.

Francisco Gil promovía el método de Robert Sutton para la inoculación ^16^. Este médico inglés perfeccionó la técnica otomana de la inoculación, la cual tenía sus orígenes en las prácticas brahmánicas de la India ^21^, y la describió así:

“[…] La lanceta cargada con la menor cantidad perceptible de materia… introdúzcala inmediatamente por punción, oblicuamente, entre la bufanda y la verdadera piel apenas suficiente para extraer sangre […]” ^5^.

Este procedimiento resultó de amplia efectividad: provocaba menos efectos adversos, era menos doloroso y permitía una inoculación rápida ^5^. La técnica usada por Mutis era distinta a la defendida por Gil. En el “Método general para curar viruelas” el sabio describía:

“[…] Lo más seguro es hacer dos incisiones de tres a cuatro líneas, una en el brazo y otra en la pierna opuesta, poniendo un pedazo de hilo de igual longitud bien pasado por la materia…se debe cubrir la herida con cualquier emplasto pegante [...]” ^12^,

en tanto que no existe documentación que describa la técnica usada por Nariño en sus procedimientos médicos.

En su disertación, Gil hace una precisa y extensa narración de la historia natural de la enfermedad, con los tratamientos que consideraba indicados en cada estadio de la infección ^16^. Es llamativa la recomendación de métodos como las sangrías, la utilización de opio para paliar los síntomas, el uso de sustancias vejigantes en los estadios finales, el mantenimiento de la salivación con agua nitrada y gargarismos con agua de limón, la administración de quina para la fiebre y el uso de cantárida (veneno secretado por algunos coleópteros) para promover la aparición de las lesiones en las primeras etapas de la infección ^16^.

El segundo libro encontrado en la biblioteca de Nariño que aborda el tema de la viruela se denomina “Disertación sobre la inoculación de las viruelas”, obra que no registra autor ni fecha de redacción ^1^. Según algunos académicos, este título, mencionado por Moreno, *et al.*, podría corresponder a la “Disertación sobre la inoculación de las viruelas” de Bonifacio Jiménez de Lorite, médico sevillano que publicó su obra en 1758 ^21^. Jiménez de Lorite defendía la implementación de la variolización en España y proponía un método similar al establecido por Sutton ^22^. Sin embargo, no es improbable que el libro de Nariño haya sido una traducción de la obra francesa “*Memoire sur l’Inoculation de la Petite Verole”* escrita por Charles-Marie de La Condamine, que, coincidencialmente, fue traducida por Jiménez de Lorite. La traducción al castellano de esta obra se publicó en 1675 y avivó aún más la intensidad del debate sobre la benevolencia de la variolización. La Condamine sufrió de viruela en un viaje a las Américas, lo que marcó profundamente su obra científica ^23^. En la obra mencionada, el naturalista y geógrafo francés hizo una intensa defensa de la variolización con especial énfasis en su adopción en las colonias. Afirmaba que desde 1743 los esclavos negros del Paraná eran inoculados por sus amos con excelentes resultados ^23^. Señalaba, asimismo, que la viruela se había ensañado con la población indígena y era responsable de más muertes que el mismo hierro español ^23^. También, describió el amplio uso del curare por parte de los aborígenes del Amazonas para atender las lesiones de la viruela ^23^.

El tercer libro sobre la viruela en posesión de Nariño era la “Disertación físico-médica, en que con la razón, autoridad y experiencia se demuestra la utilidad y seguridad de la inoculación de las viruelas” ^1^*.* Este libro pertenecía originalmente a Sebastián López Ruiz, médico y naturalista panameño que mantuvo una aguda disputa con José Celestino Mutis por el descubrimiento de la quina ^24^. López Ruiz arribó a Santa Fe en 1667 como médico y secretario del virreinato ^24^, y allí se dedicó a dictar clases particulares de matemáticas y francés ^24^^,^^25^. Nariño fue uno de sus grandes alumnos. Cuando López Ruiz fue nombrado Botánico de la Real Orden en Madrid, dejó en Santa Fe algunos de sus libros y muebles a cargo de su esposa, doña María Begoña Aldana, quien, a su vez, dejó al cuidado de Nariño las pertenencias de su esposo ^25^^,^^26^, y este las embargó por una presunta deuda que López Ruiz tenía con él ^25^^,^^26^. Este hecho es atestiguado por un expediente de 1797 en el que López Ruiz demandó la devolución de sus bienes ^26^.

Este tercer libro fue escrito originalmente en lengua toscana por el médico gaditano Juan Espallarosa, fue traducido al castellano en 1767 ^27^ y sus dos partes fueron publicadas en distintas fechas. En la primera, Espallarosa rescata la labor de los médicos griegos Pilarini y Timoni, quienes iniciaron en Constantinopla la transmisión de las técnicas orientales de variolización ^27^, así como la labor de Lady Mary Wortley Montagu. El gaditano inicia su defensa de la variolización rescatando la obra de La Condamine y poniendo de manifiesto los estragos de la enfermedad en las poblaciones amerindias de regiones como la selva brasilera ^27^. Además, narra la gran mortandad de la peste en las regiones de la Toscana, en Francia, en Levante, en Germania y en otras diversas naciones europeas ^27^. A continuación, hace una súplica a los ministros de gobierno para considerar los casos de Inglaterra, Ginebra y Holanda, sitios donde la variolización se popularizaba con notables resultados ^27^. En la segunda parte, da respuesta a las diversas objeciones contra el procedimiento y a los argumentos de diversos académicos y eclesiásticos ^27^, describe los efectos adversos, da indicaciones para su tratamiento ^27^ y, por último, cierra la obra indicando cómo se realiza el procedimiento de variolización ^27^.

Llama la atención que, en la demanda de López Ruiz para la devolución de sus libros, se exigió el retorno de la obra de Francisco Gil titulada “Preservación de las viruelas” ^25^, nombre que parece ser una abreviación del título completo del libro ya comentado: “Disertación físico-médica en la cual se prescribe un método seguro para preservar a los pueblos de viruelas”*.* De la suerte de este tomo no se sabe nada.

Con estas tres obras en su posesión y con el conocimiento de otras tantas, gracias al favor de maestros como Mutis, quien seguramente le prestó a su discípulo libros como “*Avis au peuple sur sa santé*”, tratado de medicina que contiene información sobre la viruela, muy seguramente Nariño ejerció la medicina y prestó sus servicios a las poblaciones más vulnerables ^1^.

## La necesidad de replicar la vacuna

El descubrimiento de la vacuna contra la viruela por Edward Jenner en 1796, abrió nuevas perspectivas a la ciencia en la lucha contra esta devastadora enfermedad. El conocimiento de la vacuna, muy posiblemente, llegó a la Nueva Granada con la publicación del “Tratado sobre la vacuna” de Diego de Bances y con la traducción que Pedro Hernández hizo del libro de François Chaussier “Origen y descubrimiento de la Vacuna” ^5^^,^^28^. Una de las primeras solicitudes para que la España peninsular enviara la vacuna a las Américas, provino del ayuntamiento de Santa Fe ^5^^,^^15^^,^^29^. El virrey de la Nueva Granada, Pedro Mendinueta y Múzquiz, intentó importar de Europa linfa bovina para fabricar la vacuna, sin embargo, las muestras llegaban al continente inutilizables ^30^. El médico Francisco Javier de Balmis propuso al rey Carlos IV un método para transportar la vacuna contra la viruela desde la península a las colonias: formar una cadena de niños que, mediante inoculaciones sucesivas, mantuviera la vacuna activa ^30^^,^^31^. La primera de estas expediciones traía a 20 niños y significó una revolución científica para las colonias ^32^. Entre tanto, Mutis buscaba la linfa vacunal entre los bovinos y equinos de las proximidades de Santa Fe, con el fin de fabricar su propia vacuna ^5^. La búsqueda fue infructuosa, dado que los reservorios del virus de la viruela del caballo (sustrato fundamental de la vacuna) eran animales endémicos de Europa. Intentó, además, inocular viruela humana en vacas sin obtener resultados satisfactorios ^5^^,^^32^. En este contexto, Nariño fue encarcelado y comenzó a purgar su condena en condiciones deplorables, pero recibía atención médica de su maestro José Celestino Mutis, quien lo apoyó y le brindó los recursos para continuar sus labores científicas en prisión. En la biblioteca virtual de la Universidad Nacional, se encuentra la carta que Antonio Nariño dirigió al virrey afirmando haber inventado su propia vacuna efectiva contra la viruela:

“[…] después de 47 días de trabajo, en que me han salido infructuosas varias experiencias, tengo hoy la satisfacción de presentar a vuestra excelencia un muchacho, en quien ha prendido un grano con todas las apariencias de verdadera vacuna, habiéndose seguido todos los períodos y síntomas que prescriben las dos únicas recetas que han llegado a esta capital, estando hoy precisamente en el día nono de la vacunación […]” ^26^.

Nariño afirmaba haber conseguido la vacuna con resultados idénticos a los descritos por los tratados que pululaban por Santa Fe y que describían las recetas usadas en Europa para fabricar el compuesto a partir de la linfa bovina ^1^^,^^26^. Se desconocen los ingredientes que usó Nariño para alcanzar su cometido, así como el antígeno empleado en la preparación de la supuesta vacuna. Ante el fracaso de Mutis para obtener el sustrato fundamental en el Reino, los naturalistas y médicos abandonaron la búsqueda de linfa bovina y equina ^5^. No existe registro alguno de la forma de preparación del compuesto que, supuestamente, Nariño inoculó a su sobrino. Resulta sumamente improbable que, en las paupérrimas condiciones carcelarias de Nariño, se lograra la creación de una vacuna, y más en ausencia del único compuesto conocido en ese tiempo capaz de producir inmunidad sin recurrir a la variolización o el contagio. Efectivamente, la mayor duda surge ante la ausencia en el Reino del insumo esencial para conferir valor biológico al compuesto. Lo más probable es que Nariño no hallara solución para dicho inconveniente y nunca inventara una vacuna, y que la lesión producida en su sobrino (cuya naturaleza etiológica nos es desconocida) le pareciera similar a las descritas en los libros.

No se ha documentado ninguna vacuna creada en las Indias y tampoco se ha registrado un intento exitoso distinto al de Balmis para inmunizar a los americanos en aquella época. La primera expedición de Balmis arribó a Puerto Cabello el 30 de marzo de 1803 ^5^^,^^30^. En Caracas, la expedición se dividió en dos grupos, uno de los cuales alcanzó el Reino de la Nueva Granada ^30^^,^^31^. La segunda expedición vacunal a cargo del doctor Salvany, compañero de Balmis, llegó a Santa Fe el 18 de diciembre y fue recibida por el virrey Amar y Borbón y el propio Mutis ^5^. Se desconoce lo acontecido después del intento de Nariño y lo más probable es que un fracaso sea la causa del profundo silencio histórico al respecto, pero lo verdaderamente rescatable es su intento de dar respuestas de salud pública en medio de su tragedia personal y a pesar de las condiciones de su reclusión.

## Conclusión

En las colonias americanas, la devastación generada por la viruela y los altos costos económicos, demográficos y sociales, llevaron a que la labor científica en salud se desarrollara en torno a las medidas que contuvieran el virus. La variolización, a pesar de sus detractores, tuvo un amplio impacto y una mediana aceptación entre los ilustrados de la Nueva Granada, que veían en el método un acto de responsabilidad estatal. La vacuna fue una respuesta providencial a un problema de salud pública cardinal en el continente americano. Sin embargo, en el inicio de su propagación, surgieron problemas en las dinámicas de distribución que retrasaron la llegada de la inmunización a América. Esto obligó a los ilustrados, entre ellos a Nariño, a buscar formas de replicar la vacuna en las Indias. A pesar de la ausencia de recursos, Nariño intentó inventar una vacuna y eso, independientemente de su factibilidad, demuestra su gallardía e ingenio. Los estudios de Nariño revelan su profundo humanismo, encarnado en su intento de garantizar los derechos de los ciudadanos americanos, no solo en temas políticos y sociales, sino también, en lo concerniente a su salud y bienestar.
